# The Future of Anaerobic Digestion: Challenges and Opportunities

**DOI:** 10.3390/bioengineering12050524

**Published:** 2025-05-15

**Authors:** Marcell Nikolausz, Peter Kornatz

**Affiliations:** 1 Department of Microbial Biotechnology, Helmholtz Centre for Environmental Research–UFZ, 04318 Leipzig, Germany; 2 Department of Biochemical Conversion, Deutsches Biomasseforschungszentrum Gemeinnützige GmbH (DBFZ), Torgauer Strasse 116, 04347 Leipzig, Germany; peter.kornatz@dbfz.de

Research on anaerobic digestion (AD) is still booming in the first quarter of the 21st century. According to our literature search, the number of publications related to AD has increased continuously from the turn of the century and peaked in 2021 with 3554 AD-related papers according to the Web of Science database (Figure 1a). One may ask why this topic, dealing with biomass degradation under anoxic conditions, is still so attractive among scientists. Considering the dark foul-smelling viscous slurry of AD bioreactors and similarly unattractive waste substrates such as manure, municipal waste, and various wastewater types, this question is even more valid. However, we cannot deny that there is a beauty in the complex syntrophic microbial interactions converting wastes to valuable products, or in the caviar-like structure of the granular sludge formed during anaerobic wastewater treatments and the complex microbial networks of the biofilms growing on anaerobic membranes. It is not a surprise that they still amaze scientists across the globe. We can thus conclude that the popularity of this topic is undiminished, and with the advance of novel methods, especially in the case of microbial community analyses by molecular techniques, our knowledge about AD has been increasing dramatically. Once considered a black box process, where only the input and output and a few other parameters were known, today, it is at least grey with several knowledge milestones achieved, but our understanding of AD is far from complete. Amplicon sequencing has become a standard technique for analyzing the microbial community structure and dynamics, and even a curated AD-specific 16S rRNA gene database, MiDAS (Microbial Database for Activated Sludge, https://www.midasfieldguide.org), is available to help achieve more accurate analyses of our amplicon sequencing data [1]. Similarly, the application of metagenomics approaches is not that unique anymore, and due to the technological revolution in DNA sequencing and advanced computational approaches, the rapid reconstruction of genomes is possible, as discussed in a recent review [2]. Other omics techniques, such as metatranscriptomics [3] and metaproteomics, are being applied more and more frequently in this field [4,5]. The domain Archaea, once comprising only two phyla, namely Crenarchaeota and Euryarchaeota (including methanogens), has been extended by about 30 new phyla [6], and this number will probably grow in the future. However, most of these new phyla have no cultivated members [7], and we can infer their physiology only based on genes and pathways reconstructed from metagenome-assembled genomes (MAGs).

Looking at the domain Bacteria in the field of AD, we can see a similar situation with ever-increasing numbers of new taxa described only by genes, genome sequences, and MAGs. A good example is the candidate phylum Cloacimonetes (WWE1), frequently found in anaerobic wastewater treatment systems [8] and biogas reactors [9], and its members are proposed to play important roles in syntrophic propionate oxidation and/or in cellulose degradation [10,11], but despite its predominance in AD reactors, no pure cultures are in hands of microbiologists so far.

Based on bibliometric analyses of AD-related papers published in this century examining the network of user-defined keywords, we can conclude that the major topics of this field were related to the diversification of the feed substrates, process enhancement or mitigation of process inhibition (Figure 1b). The major keywords refer to the following: (i) substrates of AD process: biomass, organic waste, manure, rice straw, wastewater, (waste-)activated sludge, municipal solid wastes, sewage sludge; (ii) products: biogas, energy, methane; (iii) process steps: hydrolysis, degradation, fermentation, methane production; (iv) process parameters and properties: organic loading rate, temperature; (v) terms related to process inhibition and enhancement: ammonia, ammonia inhibition, inhibition, (alkaline) pretreatment, aerobic digestion (Figure 1b).

**Figure 1:**
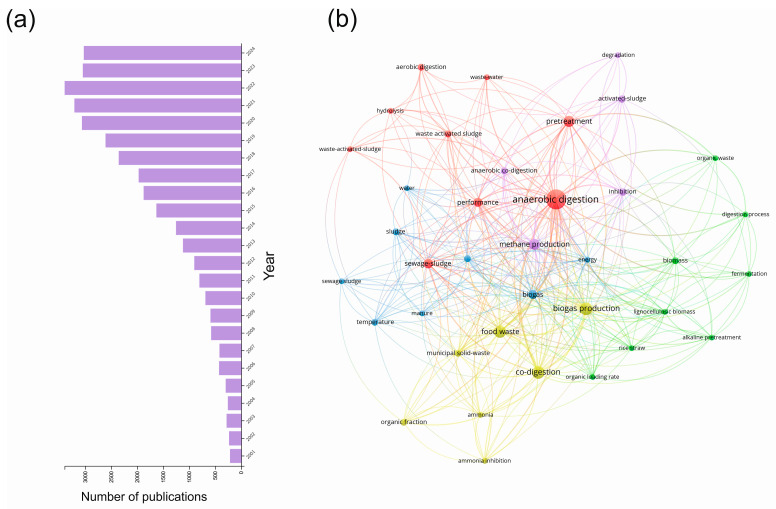
(**a**) The number of AD-related publications in the first quarter of the 21st century according to a search in the Web of Science (Clarivate) database using the term “anaerobic digestion” in the topic field (searching in title, abstract, keyword plus, and author keywords). (**b**) Bibliometric investigation showing the link among most popular keywords created by VOSViewer co-occurrence analyses among author keywords in the exported information [12] of the AD-related papers published between 1 January 2000 and 31 March 2025 (access of the data).

We have witnessed the improvement of the biochemical methane potential (BMP) assessment [13], partially due to the development of commercial batch BMP reactor systems automatically measuring methane production. However, similar relatively affordable continuously fed systems are missing; therefore, the process optimization is rarely performed by using several parallel, (semi)continuously fed reactors as biological replicates. This makes it difficult to draw ecologically relevant conclusions or prove the statistical significance of the results. Especially for the investigation of anaerobic wastewater treatment systems, laboratory-scale UASB reactors are missing [14]. The interpolation of laboratory-scale results to large-scale application has rarely been investigated [15], and the industry requires more pilot-scale studies for proving the applicability of the laboratory results in commercial systems.

In this Special Issue, two papers address pilot studies. Pyykkönen et al. used pilot- and farm-scale leach bed reactors to investigate the methane production from whole crop fava bean and horse manure, as well as the co-digestion of clover-grass silage, chicken manure, and horse manure. The authors also highlighted the importance of the management and proper storage of the digestate to minimize nitrogen loss and greenhouse gas emissions [16].

Biogas as a gaseous product of AD contains, besides methane and carbon dioxide, trace amounts of corrosive and highly toxic hydrogen sulfide. Lenis and co-workers developed a pilot-scale biotrickling filter reactor for biogas desulfurization using agricultural digestate as inoculum and biotrickling liquid providing the microorganisms and also the nutrient source for the process [17]. Furthermore, they investigated the effect of process-related fluctuations on the efficiency, and they also studied the adaptation of the microbial community and showed the co-existence and adaptation of both aerobic and anaerobic bacteria to the fluctuating operating conditions. This study is a good example of utilizing locally available bio-resources for process development and the importance of selection of proper inoculum. The start-up of a system begins with inoculation, which is in the focus of many studies in general and of three studies in this Special Issue. Cattle are considered major contributors of methane emission [18], and methanogenesis mainly happens in the rumen, as a secondary microbial activity besides the production of volatile fatty acids (VFAs) [19]. Cattle manure that contains, besides the feces, bedding material and urine collected from the stables still harbors methanogens in large abundance; therefore, it is also considered an excellent inoculum for the AD process. On the other hand, methanogens are absent or present in low abundance in the feces of many other animals, such as poultry and pig, which are still treated via AD. Wi and colleagues studied the application of dairy cattle manure as an inoculum source for the anaerobic digestion of pig manure using solid container submerged laboratory-scale reactors under mesophilic conditions. They also optimized the inoculum-to-substrate ratio and analyzed and compared the microbial communities in the inoculum and substrate [20].

The effect of microbial diversity of four inocula on the process stability, VFA formation, and methane production was investigated by Logroño and co-workers using ex situ hydrogen biomethanation in batch systems [21]. The conversion of surplus electricity to storable energy carriers, including methane and commodity chemicals and even food and feed technologies, are discussed under the umbrella term of ‘Power-to-X’ (P2X) [22]. The biomethanation of hydrogen and carbon dioxide is an important P2X technology with an increasing number of pilot- and full-scale applications. Many of these reactors use complex microbiota as inoculum, which have not yet been extensively investigated. Logroño et al. found that highly diverse inocula outperformed those of lower diversity, in which even transient VFA accumulation was observed. This advantage of the diverse inocula was attributed to the higher number of microbial functions responsible for a more stable and balanced process [21].

The objective of the study by Rajagopal et al. [23] was to investigate start-up strategies for two dry AD systems treating pig manure at low temperatures. They found that a two-stage AD system with a percolate recirculation mode of operation was far superior to the static mode, and the usage of inoculum adaptation was important to improve the methane yield, which further highlights that using a proper inoculation strategy is fundamental for the effective operation of biogas reactors [23].

The proper storage of the substrate is a challenge for the full-year operation of biogas plants. Ensiling, a traditional technique used for the preservation of plant biomass in the livestock industry, is frequently used for this purpose. Ensiling is performed via lactic acid bacteria converting water-soluble carbohydrates into organic acids, mainly lactic acid and acetic acid, which create conditions inhibiting the activity of unwanted microorganisms and preserving the substrate. Nazar and colleagues investigated the epiphytic microbiota of various forages during ensiling of sorghum. They observed differences in fermentation characteristics, which can be explained by differences in the epiphytic microbiota of the investigated plants, further highlighting the importance of inoculation in this anaerobic fermentation process [24].

The production of short- and medium-chain carboxylic acids with a higher economic value compared to biogas is an alternative future path and a potential for repurposing existing biogas plants. Such a process can be economical if cheap substrates are used, mainly side and waste products of agriculture. Pereira et al. optimized the acidogenic fermentation of spent coffee grounds and investigated the effect of various pretreatment strategies, and the best results were obtained with acidic hydrolysis [25].

The studies chosen to feature in this Special Issue cover just a small but vital section of the diverse topics in the contemporary AD literature and also highlight the enormous potential of AD to contribute to the future bioeconomy. We anticipate that AD will play a smaller and smaller role in energy carrier production over time, especially in land-intensive bioenergy production, due to land constraints, the risk of biodiversity reduction, and progress in competing technologies [26]. The future of AD also depends on policy makers, who will hopefully choose a good stewardship of biomass. We assume there will be further growth in the solid waste and wastewater treatment sector, both regarding the better penetration of anaerobic technologies and the increased role of AD in the treatment of sewage and waste-activated sludge. Moreover, a better integration of AD into the circular economy is expected via a broader product spectrum including carboxylates, polymers, and even proteins from biogas.

Overall, at the end of the first quarter of the 21st century, AD research is more dynamic than ever, and its baffling complexity still holds an enormous treasure trove of information to be explored by future generations of AD scientists.

## Abbreviations

The following abbreviations are used in this manuscript:

AD	Anaerobic digestion
VFA	Volatile fatty acids
BMP	Biochemical methane potential
MAG	Metagenome-assembled genomes
UASB	Upflow anaerobic sludge blanket
